# Expression of the sodium potassium chloride cotransporter (NKCC1) and sodium chloride cotransporter (NCC) and their effects on rat lens transparency

**Published:** 2010-05-04

**Authors:** K.N. Chee, I. Vorontsova, J.C. Lim, J. Kistler, P.J. Donaldson

**Affiliations:** 1 Department of Optometry and Vision Science, University of Auckland, Auckland, New Zealand; 2 New Zealand National Eye Centre, University of Auckland, Auckland, New Zealand; 3 School of Biological Sciences, University of Auckland, Auckland, New Zealand

## Abstract

**Purpose:**

To characterize the expression patterns of the Na^+^-K^+^-Cl^-^ cotransporter (NKCC) 1 and NKCC2, and the Na^+^-Cl^-^ cotransporter (NCC) in the rat lens and to determine if they play a role in regulating lens volume and transparency.

**Methods:**

RT–PCR was performed on RNA extracted from fiber cells to identify sodium dependent cotransporters expressed in the rat lens. Western blotting and immunohistochemistry, using NKCC1, NKCC2, and NCC antibodies, were used to verify expression at the protein level and to localize transporter expression. Organ cultured rat lenses were incubated in Artificial Aqueous Humor (AAH) of varying osmolarities or isotonic AAH that contained either the NKCC specific inhibitor bumetanide, or the NCC specific inhibitor thiazide for up to 18 h. Lens transparency was monitored with dark field microscopy, while tissue morphology and antibody labeling patterns were recorded using a confocal microscope.

**Results:**

Molecular experiments showed that NKCC1 and NCC were expressed in the lens at both the transcript and protein levels, but NKCC2 was not. Immunohistochemistry showed that both NKCC1 and NCC were expressed in the lens cortex, but NCC expression was also found in the lens core. In the lens cortex the majority of labeling for both transporters was cytoplasmic in nature, while in the lens core, NCC labeling was associated with the membrane. Exposure of lenses to either hypotonic or hypertonic AAH had no noticeable effects on the predominately cytoplasmic location of either transporter in the lens cortex. Incubation of lenses in isotonic AAH plus the NKCC inhibitor bumetanide for 18 h induced a cortical opacity that was initiated by a shrinkage of peripheral fiber cells and the dilation of the extracellular space between fiber cells in a deeper zone located some ~150 μm in from the capsule. In contrast, lenses incubated in isotonic AAH and the NCC inhibitor thiazide maintained both their transparency and their regular fiber cell morphology.

**Conclusions:**

We have confirmed the expression of NKCC1 in the rat lens and report for the first time the expression of NCC in lens fiber cells. The expression patterns of the two transporters and the differential effects of their specific inhibitors on fiber cell morphology indicate that these transporters play distinct roles in the lens. NKCC1 appears to mediate ion influx in the lens cortex while NCC may play a role in the lens nucleus.

## Introduction

Lens transparency is a direct result of its unique cellular structure; any disruption to the pseudocrystalline packing of cortical fiber cells, by either cellular swelling or dilation of the normally tight spaces between the cells, increases intralenticular light scattering [1,2]. It is therefore not surprising that lenses placed in either hypotonic or hypertonic media are capable of regulating their volume via a regulatory volume decrease (RVD) or regulatory volume increase (RVI), respectively [3-5]. Furthermore, under isotonic conditions the lens needs to actively maintain fiber cell volume to preserve overall tissue transparency [6]. This is dramatically illustrated by the histological analysis of lenses organ cultured under isotonic conditions in the presence of a variety of Cl^-^ transport inhibitors [6-10]. This analysis has revealed that blocking Cl^-^ transport induces distinctly different types of damage to fiber cells located in the lens periphery and deeper cortex indicating that distinct ion influx and efflux zones exist in the lens cortex [1]. Since these two spatially distinct ion influx and efflux pathways are coupled by gap junctions, it follows that ion and water flow in the two zones must be perfectly matched if overall lens volume and therefore transparency are to be maintained.

In a series of previous studies, we have demonstrated that the KCl cotransporter, KCC, plays a role in regulating lens volume under both isotonic and hypotonic conditions [7,9,11]. Culturing lenses in the presence of the KCC inhibitor ([dihydronindenyl]oxy) alkanoic acid (DIOA) produced a pronounced swelling of fiber cells located in the peripheral efflux zone and a dilation of the extracellular space between deeper fiber cells in the influx zone. In contrast, organ culturing lenses in the presence of a KCC activator N-ethylmaleimide (NEM) [12], caused shrinkage of fiber cells in the efflux zone, and extensive cell swelling in deeper fiber cells of the influx zone [7]. Molecular experiments have indicated that this reciprocal modulation of fiber cell volume by DIOA and NEM in the two zones is potentially mediated by three of the four known KCC isoforms which are expressed in a differentiation-dependent manner in the rat lens [7]. KCC1 was restricted to peripheral cells in the efflux zone, KCC3 was found in both zones in the outer cortex, while KCC4 was expressed throughout the entire lens including the lens core. The subcellular location of these transporters in the lens outer cortex under isotonic conditions was predominately cytoplasmic in nature, but upon exposure to hypotonic solutions KCC1 and KCC4, but not KCC3 was translocated to the plasma membrane where they presumably affect a RVD to restore lens volume.

This view was recently supported by patch clamp studies performed on fiber cells isolated from the efflux zone. These peripheral fiber cells, which normally lack the volume sensitive Cl^-^ conductance observed in longer cells from the influx zone [13], exhibited two distinctly different current responses to hypotonic challenge. While hypotonic stress caused cell swelling in both groups of short cells, in one group this was not accompanied by an increase in whole cell current, while in the other a transient increase in outward current was observed. In both groups of cells, the subsequent application of DIOA induced a large increase in outward current, indicating that inhibiting the KCC mediated ion efflux retards RVD and promotes the activation of a quiescent volume-sensitive Cl^-^ channel that mediates an ion efflux that restores fiber cell volume [11]. These experiments indicate that peripheral lens fiber cells contain parallel systems for regulating not only their steady-state volume, but respond to volume changes induced by hypotonic stress with a RVD mediated by KCC and if necessary the activation of a volume sensitive Cl^-^ channel.

The general purpose of this present study was to determine whether similar transport pathways exist in the lens that would operate in parallel to the RVD mediated by KCCs to affect a RVI that would act to restore lens volume following cell shrinkage. In other cells the coordinated activity of RVD and RVI determines the steady-state volume set point of a cell [14]. Leading candidates for the intracellular accumulation of K^+^, Na^+^, and Cl^-^ ions associated with RVI in other cells include parallel Na^+^/H^+^ and Cl^-^/HCO_3_^-^ exchangers, and the Na^+^-K^+^-2Cl^-^ cotransporter (NKCC) [15] and Na^+^-Cl^-^ cotransporter (NCC). NKCC and NCC together with the KCCs form three distinct sub-families of the electroneutral cation-chloride cotransporters (CCC) super family. In a variety of epithelial tissues, NKCC exists as two different isoforms NKCC1 and NKCC2. The “housekeeping” isoform, NKCC1 is usually located in the basolateral membranes of secretory epithelia, while NKCC2 is often found in the apical membranes of epithelial cells involved in Cl^-^ reabsorption [16,17]. Like NKCC2, NCC is largely confined to the apical membrane of absorptive epithelial cells [17,18], and like NKCC1 it is activated by cell shrinkage as well as a decrease in intracellular Cl^-^ concentration [19]. Cl^-^ transport via NCC can be distinguished pharmacologically from that mediated by NKCC due to its sensitivity to thiazide, relative to the loop diuretic bumetanide which preferentially inhibits NKCC [20].

In the rabbit lens, Alvarez et al. [21-23] have identified a baseline bumetanide sensitive Rb^+^ flux that is enhanced by hypertonic stress and the elevation of cytoplasmic cAMP levels, and which appears to be mediated at the molecular level by NKCC1 rather than NKCC2 expression. In addition, Lauf et al. [24] have used Rb^+^ and Li^+^ fluxes plus molecular techniques to identify and characterize NKCC1 and NCC expression in cultured human lens epithelia. Most recently, Bassnett and collegues [25] have used a proteomic approach to identify NKCC1 in the mouse lens. In this study, we show that NKCC1 and NCC but not NKCC2 are expressed in the rat lens epithelium and fiber cells. In addition we have used immunolabeling to demonstrate that NKCC1 and NCC exhibit different expression patterns in the rat lens cortex and core, and organ culture experiments to show that the cotransporter specific inhibitors thiazide and bumetanide differentially affect the morphology of cortical fiber cells. Our results suggest that NKCC1 and NCC play different roles in the lens cortex and core, and that together with KCC they interact to maintain steady-state lens volume and therefore transparency.

## Methods

### Chemicals

Phosphate buffered saline (PBS) tablets, [(dihydronindenyl)oxy]alkanoic acid (DIOA), fluorescein isothiocyanate-conjugated wheat germ agglutinin (FITC-WGA, *Triticum vulgaris*), tetramethyl rhodamine isothiocyanate conjugated wheat germ agglutinin (TRITC-WGA), bumetanide and thiazide were all obtained from Sigma Chemical Co. (St. Louis, MO). Primary antibodies raised against the cytoplasmic COOH-terminal tails of NKCC1 and NKCC2, and the NH_2_-terminus of NCC were obtained from Alpha Diagnostic International (San Antonio, TX). The secondary antibody, goat anti-rabbit conjugated Alexa 488, was obtained from Molecular Probes, Inc. (Eugene, OR). PCR primers were commercially synthesized; refer to Table 1 (Life Technologies, Gaithersburg, MD).

**Table 1:** Specific *NKCC* and *NCC* PCR primer sets.

**Isoform**	**Accession number**	**Oligonucleotide**	**Expected product size (bp)**	**Thermocycling Conditions**
*NKCC1*	AF051561	Sense, position #917; GTCACATACACTGCCGAAAG Antisense, position #1247; TCTGCGATTCCAACAACATA	350	35 cycles of: 94 °C for 30 s; 52.7 °C for 30 s; 72 °C for 30 s
*NKCC2*	RNU10096	Sense, position #2731; CCGCAATCAAAGACAACGAC Antisense, position #3219; CTAAACTGGTGACGACTCTT	508	40 cycles of: 94 °C for 30 s; 52.7 °C for 30 s; 72 °C for 30 s
*NCC*	RNU10097	Sense, position #520; TGGCTCATCATCCTGCTGTC Antisense, position #926; GGCTTTGTCCTTAGATGCTG	406	35 cycles of: 94 °C for 30 s; 65.3 °C for 30 s; 72 °C for 30 s

### Animals

All experiments used 21-day-old Wistar rats, which were sacrificed by CO_2_ asphyxiation and spinal dislocation. All animals procedures were approved by the University of Auckland Animal Ethics Committee in accordance with guidelines published by the Institute for Laboratory Animal Research. Rat lenses were extracted from eyes and placed in either sterile dimethylpryrocarconate (DMPC) treated PBS for extraction of RNA or into Artificial Aqueous Humor (AAH, 125 mM NaCl, 4.5 mM KCl, 10 mM NaHCO_3_, 2 mM CaCl_2_, 0.5 mM MgCl_2_, 5 mM glucose, 20 mM sucrose, 10 mM HEPES, pH 7.4; osmolarity 300mmol/kg) for organ culture experiments.

### RT–PCR

All procedures are essentially similar to those performed in [7]. Briefly, lenses were rolled on filter paper to remove adherent tissue, then decapsulated to remove epithelial cells that were attached to the capsule. The remaining fiber cell mass was immediately placed in RNA*later*^™^ (Invitrogen™, Carlsbad, CA) and total RNA isolation performed using the RNAqueous™ kit (Invitrogen) following the manufacturer's protocols. Total RNA from positive control tissue (kidney) was extracted using TRIzol™ (Gibco, Grand Island, NY) following standard protocols. The RNA was reverse-transcribed using the ThermoScript^™^ RT–PCR system (Invitrogen). PCR amplification was performed using a reaction mixture that contained: 2 μl of cDNA, 1× PCR buffer, 0.2 mM dNTPs, 1–2.5 mM MgCl_2,_ 2 Units Platinum® *Taq* DNA Polymerase, and 0.2 μM sense and anti-sense primers (Table 1). NKCC1, NKCC2, and NCC primers were designed to regions that were not homologous among the three closely related family members, thereby creating isoform specific PCR primer sets. After initial denaturation for 2 min at 94 °C, PCR was performed using the thermocycling conditions listed in Table 1. After a final elongation step of 72 °C for 7 min, PCR products were analyzed by electrophoresis on 0.8% agarose gels. QIAquick Gel Extraction Kit (Qiagen, Hilden, Germany) was used to extract PCR products from gels for direct sequencing.

### Western blot analysis

Crude membrane proteins were extracted from eight decapsulated rat lenses and one kidney. Tissue was homogenized in 1 ml homogenate solution (5 mM Tris, pH 8.0, 5 mM EDTA, 5 mM EGTA, and 1× protease inhibitor cocktail [Complete EDTA-free; Roche Diagnostics, Indianapolis, IN]) then pelleted in a SS34 rotor (RC 5C; Sorvall, Newton, CT) at 17,230× g for 20 min. The pellets were washed twice in storage buffer (5 mM Tris, pH 8.0, 2 mM EDTA, 100 mM NaCl, and 1× protease inhibitor cocktail) and resuspended in the storage buffer to a concentration of approximately 4 mg/ml and stored at −20 °C. Crude fiber membrane fractions from the outer cortex, inner cortex, and core of the lens were prepared by microdissecting 10 to 15 decapsulated lenses using a microscope and a pair of sharpened tweezers [26]. The resultant three fractions were then homogenized, washed, and stored as described above for the preparation of total crude fiber membranes. A BCA assay (Thermo Fisher Scientific, Rockford, IL) was performed to determine equal protein loading concentrations for each membrane preparation. Proteins (~30 µg per lane) were first separated on a 10% SDS-polyacrylamide gel and then transferred onto nitrocellulose membranes (Hybond-C; Amersham Life Science, Arlington Heights, IL) by electrophoresis for 90 min at 170 mA. Membranes were stained (1% Ponceau, 1% acetic acid in milliQ H_2_O) to confirm transfer and integrity of proteins, washed in milliQ H_2_O and then membranes were incubated overnight at 4 °C in blocking solution (1% BSA and 0.1% Tween-20 in TBS: 2 mM Tris-HCl, 140 mM NaCl, pH 7.6). Membranes were subsequently incubated for 2 h with primary antibodies diluted in tris-buffered saline (TBS; NKCC1 1:2,000; NKCC2 1:500, NCC 1:2,000). Antibody labeling of the membranes was visualized using chemiluminescence as per the manufacturer’s instructions (ECL™; Amersham Life Sciences).

### Immunohistochemistry

Organ cultured lenses were fixed in 0.75% paraformaldehyde in PBS for 24 h and cryoprotected using established procedures [27]. Subsequently, lenses were frozen in Tissue-Tek® OCT (Sakura Finetek, Torrance, CA) and 10 μm thick sections cut using a cryostat (CM3050; Leica Microsystems, Wetzlar, Germany). Sections were placed on poly-L-Lysine (Sigma) coated microscope slides and non-specific binding inhibited by incubating sections in blocking solution (3% BSA and 3% normal goat serum) for 1 h followed by washing in PBS (3× for 5 min each time). All antibodies were diluted in blocking solution. Primary antibodies (2 µg/ml) were added to the sections which were then incubated for 2 h at room temperature. Following PBS washes (6× for 5 min each time), sections were incubated with the secondary antibody, goat anti-rabbit conjugated to Alexa 488, at a dilution of 10 µg/ml. Fiber cell membranes were labeled with WGA-conjugated TRITC (diluted at 5 μl/ml in PBS) for 1 h at room temperature. Following a series of washes in PBS (6× for 5 min each time), slides were mounted using CITIFLUOR™ (Agar Scientific, Essex, UK) and sealed against moisture loss. Negative control sections were performed using identical procedures except that primary antibodies were first preincubated with a 10-fold excess of their antigenic peptide for 1 h. The subsequent treatments were identical to those outlined above. Sections were viewed using a Leica TCS SP2 confocal laser scanning microscope (Leica Lasertechnik, Heidelberg, Germany). Labeling patterns were collected separately using Leica’s SCANware software and merged using Adobe® Photoshop® 6.0.

### Lens organ culture experiments

Lenses were extracted and placed in AAH + 1% penicillin/streptomycin for 1 h at 37 °C. Lenses which developed opacities during this pre-incubation period were discarded. To assess the effect of changes in osmolarity on the subcellular distribution of NKCC1 and NCC, lenses were organ cultured in Hypotonic AAH (50 mM NaCl, 175±5 mOsmol) or Hypertonic AAH (200 mM NaCl, 425±5 mOsmol) at 37 °C for 18 h to induce cell swelling and shrinkage, respectively, and transporter location and fiber cell morphology visualized by immunohistochemistry (see above). For inhibitor experiments some 6–8 transparent lenses were transferred to either fresh AAH (control) or to AAH containing either bumetanide (2 to 100 μM), or hydrochlorothiazide (10 to 100 μM; Sigma) and incubated for up to 18 h at 37 °C. Bumetanide and thiazide were dissolved in AR grade ethanol and methanol, respectively. Lenses incubated in AAH with only the ethanol vehicle exhibited no changes in tissue architecture. At appropriate time points, lenses were fixed in 25% Karnovsky’s solution (1% paraformaldehyde, 50 mM sodium cacodylate, 1.25% glutaraldehyde, pH 7.4; osmolarity 300 mmol/kg in PBS) for 4 h at room temperature. Lens transparency was assessed with a dissecting microscope fitted with dark field optics. Fixed lenses were then gently rolled on Whatman filter paper to remove any adherent tissue. Lenses were then super glued to the plate of a vibrating knife microtome (Leica VT1000, Technical Products International Inc., St. Louis, MO) in either axial or equatorial orientation, and 170 μm thick sections cut. Sections were incubated in FITC-WGA (5 μl/ml in PBS) overnight in the dark at room temperature. After six 5-min washes in PBS, sections were mounted onto glass slides using CITIFLUOR™ and sealed with nail polish to prevent dehydration. Membrane labeling was visualized on sections using confocal microscopy.

## Results

### Molecular identification and localization of sodium-dependent chloride cotransporters in the lens

**Figure 1:**
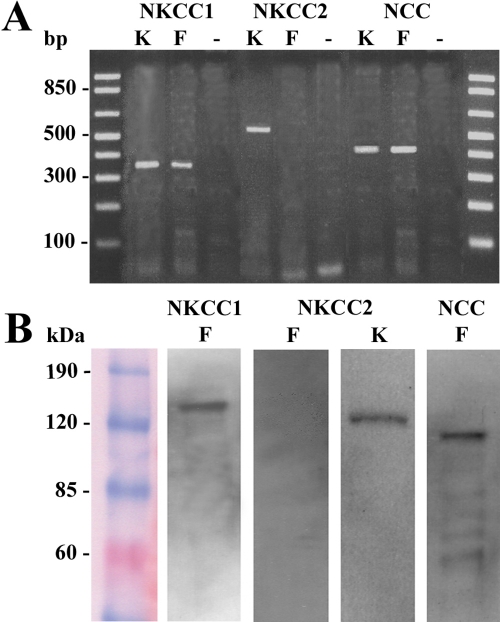
Identification of *NKCC* and *NCC* in the rat lens. **A**: Agarose gel showing reverse-transcription polymerase chain reaction (RT–PCR) products amplified using total RNA extracted from kidney (K) and lens fiber cells (F). PCR products were detected for *NKCC1* in kidney and fiber cells; for *NKCC2* in kidney but not in fiber cells; and for *NCC* in kidney and fiber cells. Negative control lanes which lacked reverse transcriptase (-) show no amplification. First and last lanes show DNA ladders for analysis of PCR product size. **B**: Western blot analysis showing the presence of NKCC1 and NCC, but not NKCC2 in crude rat lens membrane preparations. A band however, was detected for NKCC2 in the kidney thereby confirming antibody activity.

PCR conditions were optimized using the kidney as a control tissue in which all three cotransporters had been previously identified [15,20]. These optimized conditions were then used to amplify PCR products from cDNA reverse transcribed from isolated lens fiber cells (Figure 1A). PCR products of expected sizes (Table 1) were identified for *NKCC1* and *NCC*. No product for *NKCC2* was detected in fiber cells, although it was amplified from kidney. Products amplified in all tissues were verified by direct sequencing (data not shown) and no amplification products were detected in RT-negative controls.

Having identified transcripts for *NKCC1* and *NCC*, but not *NKCC2*, western blotting was used to verify this molecular profile at the protein level using isoform-specific commercially available antibodies (Figure 1B). Protein bands representing NKCC1 (~131 kDa) and NCC (~110k Da) were found in kidney and in isolated fiber cells. A band of the correct size (~121 kDa) was detected for NKCC2 in the kidney, but no band was detected in fiber cells, a result consistent with the molecular profile determined by RT–PCR (Figure 1A).

### Differential expression and localization of NKCC1 and NCC in the lens

**Figure 2:**
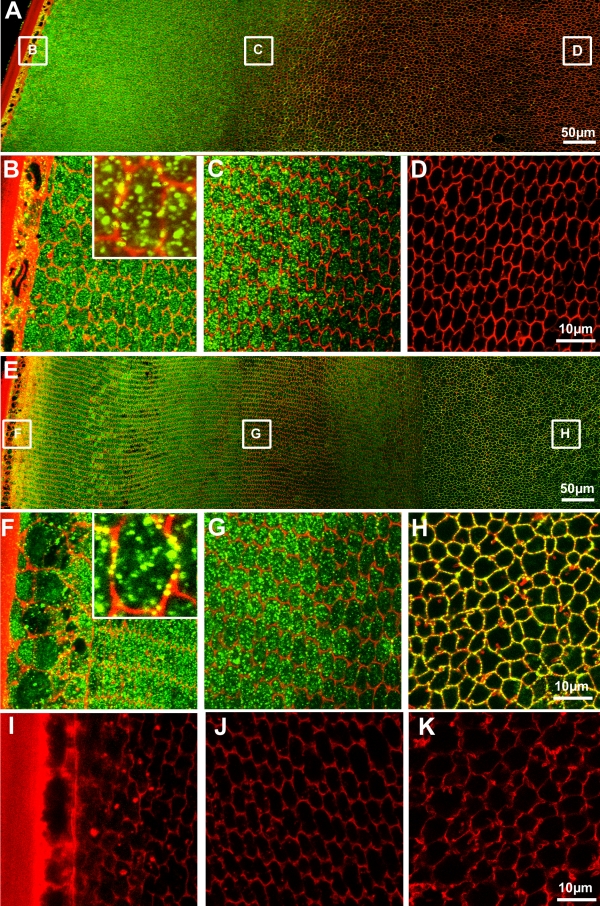
Localization of NKCC1 and NCC expression in the rat lens. Equatorial cryosections doubled-labeled with either NKCC1 or NCC specific antibodies (green), and the membrane marker TRITC-WGA (red). **A**: Image montage of the NKCC1 labeling pattern from the periphery to the core of the lens. **B**-**D**: High-powered images of areas indicated in **A**. **B**: In the lens periphery labeling for NKCC1 in the epithelial and peripheral fiber cells is predominately cytoplasmic in nature although some membrane labeling is evident (insert). **C**: NKCC1 antibody labeling is lost 350–400 μm in from the capsule. **D**: No NKCC1 labeling was found in the lens core. **E**: Image montage of the NCC labeling pattern from the periphery to the core of the lens. **F**-**H**: High-powered images of areas indicated in **E**. **F**: In the lens periphery labeling for NKCC1 in the epithelial and peripheral fiber cells is predominately cytoplasmic in nature although some membrane labeling is evident (insert). **G**: Image of the transition zone showing a shift of NCC labeling from the cytoplasm to the membrane. **H**: NCC in the core is strongly associated with fiber cell membranes. **I**-**J**: High-powered images of the outer cortex (OC), inner cortex (IC) and core (C), respectively, in which sections were labeled with the NCC antibody preabsorbed with its antigenic peptide.

Having established that both NKCC1 and NCC are expressed in the rat lens at the transcript and protein levels, we next wanted to compare their in situ localizations throughout the lens. To achieve this, equatorial cryosections were double-labeled with either NKCC1, or NCC antibodies (green; Figure 2) and the membrane marker WGA (red; Figure 2). NKCC1 was found to be expressed in the epithelium and outer cortex of the lens (Figure 2A-C), however, 350–400 µm in from the lens surface NKCC1 expression decreased in the inner cortex (Figure 2C), with no NKCC1 labeling being detected in the core of the lens (Figure 2D). In the outer cortex the majority of NKCC1 labeling was evident as punctuate labeling in the cytoplasm, although some minor labeling of the plasma membrane was observed (Figure 2B, insert). Unlike NKCC1, the expression pattern of NCC was not restricted to the epithelium and outer cortex of the lens, but extended throughout the entire lens (Figure 2E). In the outer cortex NCC was primarily located in the cytoplasm, although again a small fraction of membrane labeling was observed (Figure 2F, insert). With distance in from the lens capsule 350–400 µm there was a shift of NCC labeling from mainly cytoplasmic to more membranous (Figure 2G) and in the lens core, NCC labeling was solely associated with the plasma membranes of mature fiber cells (Figure 2H).

**Figure 3:**
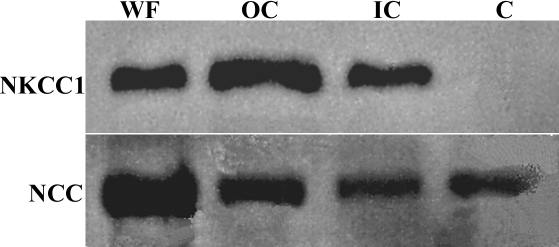
Regional expression of NKCC1 and NCC. Western blots of equal loading of crude membrane preparations from the whole lens (WF) and fractions from the outer cortex (OC), inner cortex (IC) and core (C). While both NKCC1 and NCC were present in both cortical fractions, only NCC was found in the core.

This differential expression of NKCC1 and NCC between the cortex and core was further verified by performing western blot analysis on fiber cell membrane fractions obtained from the outer cortex, inner cortex and core by microdissection (Figure 3). Consistent with the antibody labeling patterns, NKCC1 was detected in the outer and inner cortical fiber cells but not membranes from the core. Similarly bands of expected size for NCC were detected in all the lens fiber cell fractions including the core, a result consistent with its immunolabelling pattern.

**Figure 4:**
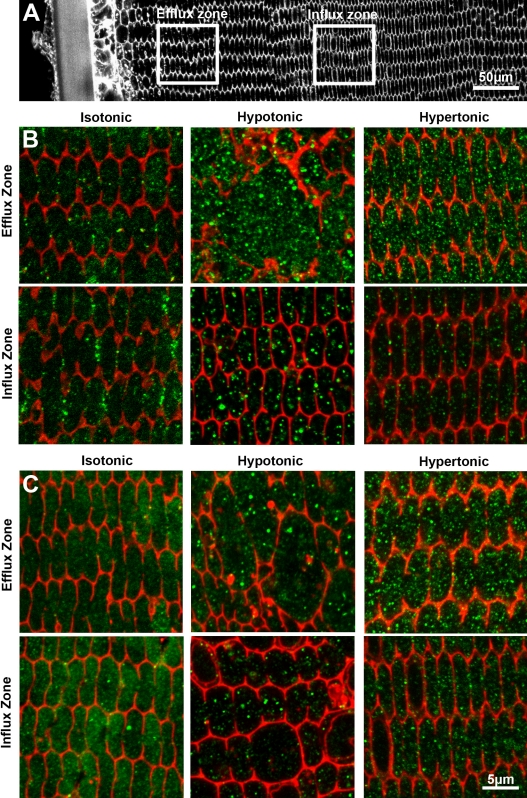
Effect of osmotic stress on the subcellular distribution of NCC and NKCC1 in the cortex of the rat lens. Images from equatorial cryosections obtained from lenses organ cultured in either isotonic, hypotonic or hypertonic AAH, and doubled-labeled with either, NKCC1 or NCC specific antibodies (green), and the membrane marker TRITC-WGA (red). **A**: Overview image of fiber cell morphology in the lens cortex taken from a lens incubated under isotonic conditions to indicate the relative locations of the efflux and influx zones. **B**, **C**: Representative high power images of NCC (**B**) and NKCC1 (**C**) labeling taken from the efflux (top panels) and influx (lower panels) zones from lenses incubated for 18 h in isotonic (left column), hypotonic (middle column), and hypertonic (right column) AAH for 18 h.

Previously, we showed that specific KCC isoforms can be recruited from a cytoplasmic vesicular pool of transporters to the plasma membrane upon exposure to hypotonic stress [7]. To investigate whether a similar recruitment of NKCC1 and NCC can be induced, lenses were organ cultured in hypotonic and hypertonic AAH and the effects on fiber cell morphology and subcellular location of NKCC1 and NCC in the lens cortex determined by immunohistochemistry (Figure 4). As characterized previously [28] culturing lenses in isotonic AAH for 18 h had no effect on fiber cell morphology (Figure 4A), but exposure to hypotonic and hypertonic AAH caused fiber cell swelling and shrinkage in the outer cortex, respectively (data not shown). To assess whether changing the extracellular osmolarity had any effect on the subcellular distribution of NKCC1 and NCC, representative high power images of antibody labeling taken from the peripheral efflux and deeper influx zones (Figure 4A). Changing the extracellular osmolarity had no obvious effect on the subcellular distribution of NKCC1 (Figure 4B) or NCC (Figure 4C) in either the efflux or influx zones of the outer cortex. For both transporters the labeling appears more punctate under hyper and hypotonic conditions, suggesting some effect of osmotic stress on the cytoplasmic pool of the transporters, but the obvious membrane insertion observed for KCC was not present [7].

### Effects of NCC and NKCC inhibitors on fiber cell morphology

**Figure 5:**
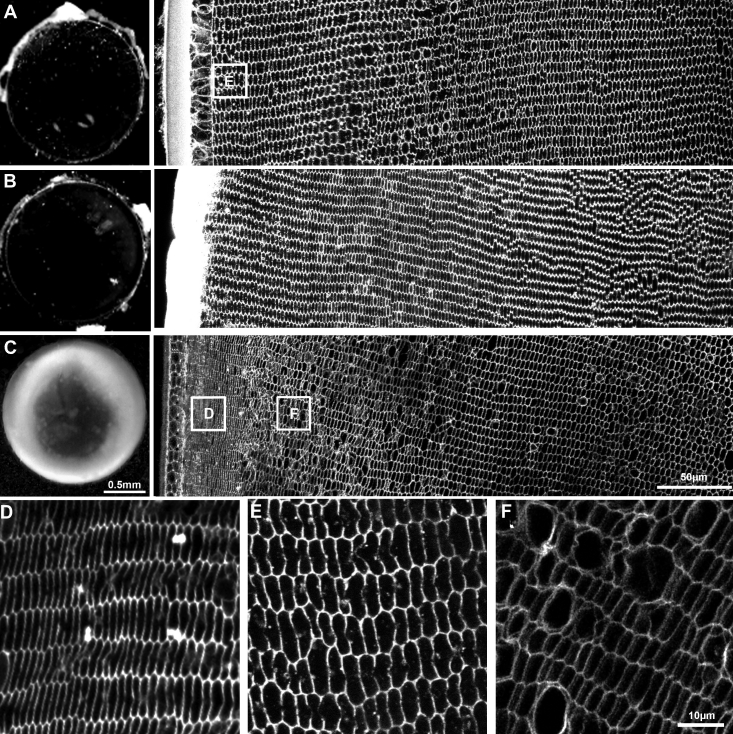
Effects of NCC and NKCC inhibitors on lens transparency and fiber cell morphology. Rat lenses were organ cultured for 18 h in isotonic AAH in either the absence (**A**) or presence of the NCC inhibitor thiazide (**B**) or the NKCC inhibitor bumetanide (**C**). Lens transparency was monitored by dark field microscopy while fiber cell morphology was determined by imaging equatorial sections labeled with the membrane marker WGA. Culturing lenses in AAH only (**A**) or AAH + 10 μM thiazide (**B**) had no major effects on lens transparency (left panels) or fiber cell morphology (right panels). **C**: Culturing lenses in AAH + 2 μM bumetanide caused cortical opacification of the lens (left panel) that was induced by damage to cortical fiber cell morphology (right panel). **D**-**F**: High powered images from the areas indicated (boxes) in **A** and **C**. Fiber cells in the lens periphery exhibited a marked shrinkage in the presence of bumetanide (**D**) relative to that observed in the absence of the inhibitor (**E**), while in the deeper influx zone dilations of the extracellular space between fiber cells (**F**) was observed in the presence of bumetanide.

To determine whether NCC and NKCC are functional in the lens cortex, we used a functional imaging approach [10] to determine whether the two transporters are active and contribute to the maintenance of steady-state lens volume in the lens cortex. Lenses were organ cultured for 18 h in isotonic AAH in the presence or absence of either thiazide (10 μM) or bumetanide (2 μΜ), which in low concentrations specifically inhibit NCC and NKCC, respectively, and have no significant effects on KCC [29-31]. Lenses incubated in isotonic AAH in the absence of the drugs remained transparent for the duration of the incubation period (Figure 5A, left panel), and cortical fiber cells maintained their regular cellular morphology when viewed using confocal microscopy (Figure 5A, right panel). Lenses organ cultured in the presence of 10 µM thiazide for 18 h also maintained their transparency (Figure 5B, left panel) and exhibited no disruption to their cellular architecture (Figure 5B, right panel), suggesting that either NCC is not actively involved in maintaining steady-state lens volume, or that the drug dose used was ineffective. To test the latter, the concentration of thiazide was increased by 10-fold however no further effects on lens transparency or fiber cell morphology were observed (data not shown) indicating that NCC does not appear to be involved in maintaining steady-state volume of cortical fiber cells.

In contrast, organ culturing lenses under isotonic conditions in the presence of 2 μM of the NKCC inhibitor bumetanide produced an intense cortical opacification (Figure 5C, left panel) which was associated with changes to the morphology of fiber cells in the lens cortex (Figure 5 C, right panel). This change in morphology was manifested as two distinct damage phenotypes. Fiber cells in the lens periphery exhibited a zone ~50 µm in from the capsule of significant compaction which at higher resolution appeared to be due to significant shrinkage of fiber cells (Figure 4D) relative to fiber cells organ cultured for 18 h in the absence of bumetanide (Figure 4E). In the deeper cortex, this compressed fiber cell morphology was replaced by a damage zone characterized by a dilation of the extracellular space between adjacent fiber cells (Figure 4F). From these inhibitor experiments it would appear that while NKCC1 contributes to the maintenance of steady-state lens volume, presumably via mediating ion influx in the outer cortex, NCC apparently does not.

## Discussion

In this current study we show that NCC and NKCC1, but not NKCC2, are expressed in the rat lens at both the transcript and protein levels, and furthermore that NCC and NKCC1 are differentially expressed throughout the lens. In epithelial and cortical fiber cells, although some membrane labeling was apparent for both NCC and NKCC1, the majority of antibody labeling for both transporters was predominately located to the cytoplasm, indicating that the majority of transporters reside as a vesicular pool of inactive transporters. At the transition between differentiating and mature fiber cells, NCC appeared to be recruited from this cytoplasmic pool to the plasma membrane as indicated by the intense membrane labeling observed for NCC in the lens core. This differentiation dependent recruitment of NCC to the plasma membrane of mature fiber cells, and the apparent absence of NKCC1 in the lens core indicates that NCC and NKCC1 may play different roles in the core and cortex of the lens. This conclusion is supported by the markedly different effects on fiber cell morphology in the lens cortex observed when lenses were organ cultured under isotonic conditions in the presence of either the NKCC1 inhibitor, bumetanide or the NCC inhibitor thiazide. The significance of these new results and how they fit into an emerging model of lens volume regulation will be discussed in turn.

The predominately cytoplasmic labeling pattern observed for both NKCC1 and NCC appears to be a reoccurring theme in lens cell biology which has been proposed to represent a dynamic vesicular pool of inactive membrane transporters that can be recruited to the plasma membrane [32,33]. In the cortex, it has been shown that other membrane transporters and ion channels located in a similar cytoplasmic pool can be dynamically recruited to the plasma membrane in response to environmental stimuli such as changes in transporter substrate availability [34] and osmotic stress [7,28]. Chee et al. [7] have demonstrated that in response to hypotonic challenge, KCC1 and KCC4 are recruited to the membranes of swollen fiber cells located in the peripheral efflux zone of the lens. More recently Suzuki-Kerr et al. [28] has shown that the ATP-gated non-selective cation channels from the P2X family also undergo an isoform specific recruitment to the membrane in response to hypotonic (P2X4 and P2X6) or hypertonic (P2X1 and P2X4) challenge. However, unlike KCC this recruitment of P2X channels occurred in the deeper ion influx zone of the lens.

In the present study osmotic stress appeared to have no major effects on the subcellular distribution of NKCC1 or NCC in either the efflux or influx zones of the lens cortex (Figure 4). This lack of recruitment to the membrane for NKCC1 and NCC was the expected result for hypotonic challenge since it is known that in response to swelling the lens elicits a RVD that is mediated by KCC and Cl^-^ channels [1], however the absence of transporter recruitment following exposure to hypertonic solutions was somewhat unexpected. The lens is known to respond to hypertonic challenge with a RVI [4], and hypertonic stress has been shown to increase bumetanide-sensitive Rb^+^ fluxes, at least in the rabbit lens [23]. So if NKCC1 and potentially NCC are involved in the RVI response to hypertonic challenge, the required increase in ion influx is probably achieved by a mechanism other than recruitment of additional NKCC1 and/or NCC transporters to the membrane. Indeed Alvarez et al. [22] have shown that in rabbit lenses organ cultured under isotonic conditions, elevation of cAMP levels enhances bumetanide-sensitive Rb^+^ fluxes, presumably by modulating the phosphorylation status of NKCC1 [18].

**Figure 6:**
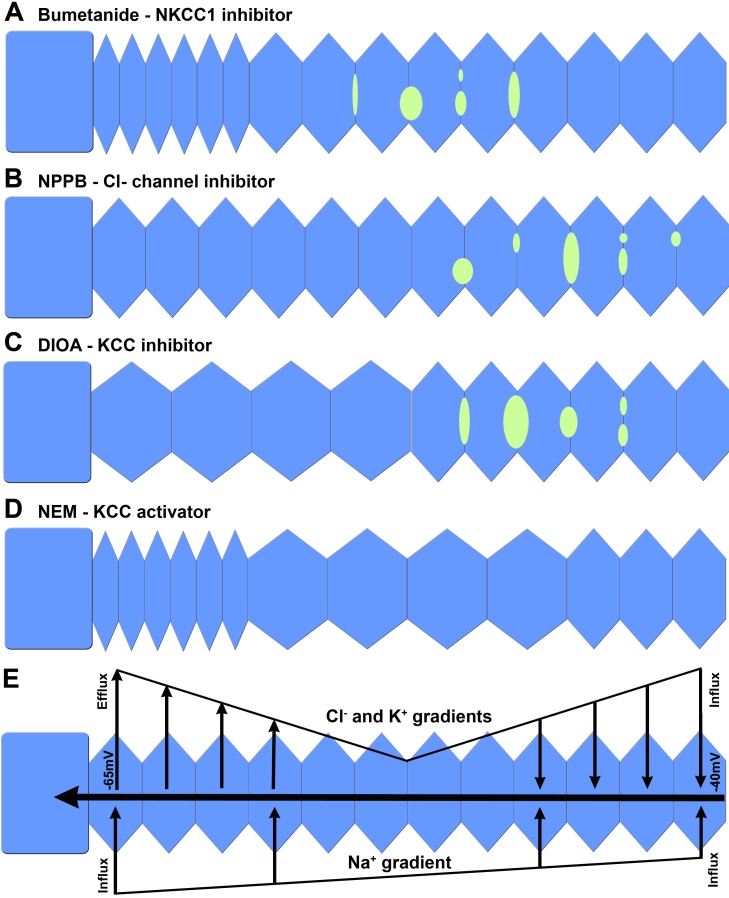
Modulation of ion transport pathways has specific effects on fiber cell morphology in the rat lens cortex. Schematic diagrams summarizing the distinctly different damage phenotypes induced in the outer cortex of rat lenses organ cultured in the presence of modulators of cation chloride cotransporters and Cl^−^ channels for 18 h. **A**: The NKCC inhibitor bumetanide (2 μM) caused peripheral cell shrinkage and extracellular space dilations in the deeper influx zone. **B**: The Cl^-^ channel inhibitor NPPB (10 μM) had no effect on peripheral fiber cells at this concentration, but did induce extracellular space dilations between deeper fiber cells. **C**: The KCC inhibitor DIOA (10 μM) caused peripheral cell swelling and deeper extracellular space dilations. **D**: The KCC activator NEM (1 mM) caused shrinkage of peripheral fiber cells but swelling of deeper fiber cells in the influx zone. **E**: The observed spatially distinct damage phenotypes can be explained by differential changes in the electrochemical ion gradients for Cl^-^ (E_Cl_) and Na^+^ (E_Na_) that occur with radial distance into the lens [40] and their effects on the direction of ion flux mediated by transporters in the two regions of the lens. In peripheral fiber cells outwardly directed Cl^-^ gradients favor Cl^-^ efflux mediated predominately by KCC [11], while in deeper fiber cells the lower transmembrane potential results in a shift in E_Cl_ that favors influx mediated by KCC and Cl^-^ channels located in this zone of the lens. In contrast E_Na_ is expected to favor ion influx throughout the entire lens cortex.

Despite the majority of NCC and NKCC1 labeling being localized to the cytoplasm there was some evidence of membrane labeling (Figure 2). However, if NCC is in the membrane, it does not appear to be a major contributor to cortical fiber cell regulation since incubation of lenses within the NCC inhibitor thiazide had no effect on fiber cell morphology (Figure 5B). In contrast, the NKCC1 membrane resident transporters produced major effects on cortical fiber cell morphology. It is conceivable therefore that NCC may be active in the outer cortex, but its inhibition by thiazide is compensated by upregulation of NKCC1 activity. However we cannot exclude the possibility that thiazide is simply not reaching its target due to penetration problems. Although this seems unlikely, since bumetanide, a structurally related compound exerted visible effects in the deeper cortex. The bumetanide specific damage phenotype consisted of both cell shrinkage in peripheral fiber cells and extracellular space dilations in the deeper ion influx zone, a unique combination of damage phenotypes that has not been observed using other modulators of cation chloride cotransporters and/or chloride channels (Figure 6). However, the ability of bumetanide to induce extracellular space dilations in the deeper cortex was similar to that observed by incubating lenses in the presence of either the Cl^-^ channel blocker NPPB [10], or the KCC inhibitor DIOA [7], suggesting that NKCC1 contributes to ion influx in this region of the lens. This is to be expected since it is the inwardly directed electrochemical gradient for Na^+^ (Figure 6E) that primarily determines the direction of NKCC1 mediated ion transport [23]. In contrast, the cell shrinkage observed in the efflux zone is hard to explain on the basis of NKCC1 inhibition alone. A similar shrinkage of fiber cells was observed upon incubation of lenses with the KCC activator NEM [7]. The similarity between the damage phenotypes induced by bumetanide and NEM tends to suggest a balance between NKCC1 mediated ion influx and KCC mediated ion efflux exists in these peripheral fiber cells. Our data suggests that blocking the NKCC1 mediated ion influx with bumetanide allows KCC mediated ion efflux to dominate causing the observed cell shrinkage. Hence using our functional imaging approach, we have independently confirmed the earlier work of Alvarez et al. [22,23] and shown that NKCC1 mediates ion influx in the lens cortex which is important for the maintenance of normal lens transparency (Figure 5C). However, determining whether NKCC1 is also involved in mediating RVI and the elongation of fiber cells in the outer cortex as suggested by Alvarez et al. [22,23] will require further experimentation.

The apparent loss of NKCC1 labeling in the core of the lens and the recruitment of NCC to the plasma membrane appeared to occur at a specific stage of fiber cell differentiation where differentiating fiber cells lose their nuclei and become mature fiber cells. We have proposed that this differentiation dependent insertion is required to change the membrane protein expression profile of mature fiber cells in the lens core that are rendered incapable of de novo protein synthesis by the loss of cell nuclei and other cellular organelles [35-37]. The regional differences observed in both NKCC1 and NCC expression patterns and the differential effects of bumetanide and thiazide on cortical fiber cell morphology tend to indicate a switch in the transporter responsible for Na^+^ dependent uptake of Cl^-^ from NKCC1 in the cortex to NCC in the lens core. Interestingly, a similar change in the expression pattern of KCC was observed in the cortex relative to the core [7]. While KCC1, KCC3, and KCC4 were all expressed in the outer cortex, only KCC4 exhibited recruitment to the membranes of mature fiber cells upon the loss of cell nuclei. While the reasons why NCC and KCC4 are the preferred cation chloride cotransporters in the core are not readily apparent, but may be due to the more acidic and hypoxic environment in the core relative to the cortex [7,38,39]. It would appear that they may work in parallel to mediate NaCl and KCl uptake in the lens core. Unfortunately attempts to rigorously test this interpretation of our data will require measurements of NCC and KCC4 activity in the lens core which are currently hampered by an inability to isolate mature fiber cells from this region of the lens.

In conclusion, our study has confirmed the presence of NKCC1 in the rat lens and has added NCC to a growing inventory of transporters and channels involved in effecting changes in fiber cell volume, under both steady-state and aniosmotic conditions, which ultimately control overall lens transparency [1]. In other tissues, the activity of these cation chloride cotransporter family members are regulated by changes in the phosphorylation status of these transporters. Future work will investigate manipulating endogenous regulatory pathways to determine whether modulating transporter activity affords protection against cortical cataract.
